# Comparison of metal-dependent catalysis by HIV-1 and ASV integrase proteins using a new and rapid, moderate throughput assay for joining activity in solution

**DOI:** 10.1186/1742-6405-6-14

**Published:** 2009-06-29

**Authors:** Mark D Andrake, Joseph Ramcharan, George Merkel, Xue Zhi Zhao, Terrence R Burke, Anna Marie Skalka

**Affiliations:** 1 Institute for Cancer Research, Fox Chase Cancer Center, 333 Cottman Avenue, Philadelphia, PA 19111, USA; 2 Locus Pharmaceuticals, Inc, Blue Bell, PA, USA; 3 Laboratory of Medicinal Chemistry, Center for Cancer Research, National Cancer Institute, Frederick, MD 21702, USA

## Abstract

**Background:**

HIV-1 integrase (IN) is an attractive target for the development of drugs to treat AIDS, and inhibitors of this viral enzyme are already in the clinic. Nevertheless, there is a continuing need to devise new approaches to block the activity of this viral protein because of the emergence of resistant strains. To facilitate the biochemical analysis of wild-type IN and its derivatives, and to measure the potency of prospective inhibitory compounds, a rapid, moderate throughput solution assay was developed for IN-catalyzed joining of viral and target DNAs, based on the detection of a fluorescent tag.

**Results:**

A detailed, step-by-step description of the new joining assay is provided. The reactions are run in solution, the products captured on streptavidin beads, and activity is measured by release of a fluorescent tag. The procedure can be scaled up for the analysis of numerous samples, and is substantially more rapid and sensitive than the standard radioactive gel methods. The new assay is validated and its utility demonstrated via a detailed comparison of the Mg^++^- and Mn^++^-dependent activities of the IN proteins from human immunodeficiency virus type 1 (HIV-1) and the avian sarcoma virus (ASV). The results confirm that ASV IN is considerably more active than HIV-1 IN, but with both enzymes the initial rates of joining, and the product yields, are higher in the presence of Mn^++ ^than Mg^++^. Although the pH optima for these two enzymes are similar with Mn^++^, they differ significantly in the presence of Mg^++^, which is likely due to differences in the molecular environment of the binding region of this physiologically relevant divalent cation. This interpretation is strengthened by the observation that a compound that can inhibit HIV-1 IN in the presence of either metal cofactors is only effective against ASV in the presence of Mn^++^.

**Conclusion:**

A simplified, assay for measuring the joining activity of retroviral IN in solution is described, which offers several advantages over previous methods and the standard radioactive gel analyses. Based on comparisons of signal to background ratios, the assay is 10–30 times more sensitive than gel analysis, allows more rapid and accurate biochemical analyses of IN catalytic activity, and moderate throughput screening of inhibitory compounds. The assay is validated, and its utility demonstrated in a comparison of the metal-dependent activities of HIV-1 and ASV IN proteins.

## Background

Retroviral integrase (IN) catalyzes the insertion of a duplex DNA copy of the viral RNA genome into the DNA of its host cell. This process establishes the retroviral provirus as a permanent component of the host cell genome, and is required for normal viral gene expression via host cell components. IN proteins are members of a superfamily of polynucleotidyl transferases, which include transposases and other recombinases. The HIV-1 IN is of special interest as a target for the development of drugs to treat AIDS [1]. For both medical and scientific reasons therefore, the biochemistry of IN proteins has been the focus of intense investigation.

**Figure 1:**
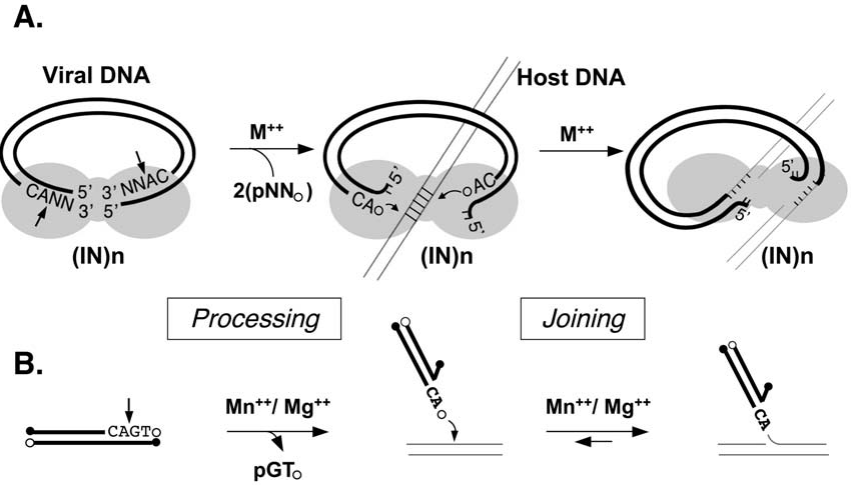
**The retroviral DNA integration reaction**. Panel A. The processing and joining steps catalyzed by retroviral integrases produce a gapped recombination intermediate. The shaded region represents an IN multimer, heavy lines the viral DNA, and thin lines host DNA. The position of the conserved CA dinucleotides at the ends of the viral DNA is shown and the position of the processing cleavage sites are marked with straight arrows. The curved arrows indicate the staggered phosphodiester bonds cleaved during the joining reaction. Panel B. Simple *in vitro *assays for IN activity represent reactions at a single viral DNA end. Viral(donor) or host(target) DNAs are distinguished as in A. Filled circles mark the 5' phosphate ends and open circles the 3' hydroxyl ends.

IN proteins catalyze two sequential and temporally distinct reactions during infection, see (Figure 1A) [2,3]. In the first reaction, called processing, two nucleotides adjacent to a conserved CA dinucleotide are removed from the 3' end of newly synthesized viral DNA. The sequence near the viral DNA ends determines the specificity for cognate viral IN proteins. The processing reaction can take place in the cytoplasm before the complex of viral DNA and IN gains access to host DNA in the nucleus. Following nuclear entry, the newly processed 3' ends of the viral DNA are joined by IN to staggered sites on both strands of the host DNA in a concerted cleavage and ligation reaction. The joining reaction produces gaps in the host DNA adjacent to the 5' ends of the viral DNA. The damage incurred by formation of this intermediate is then repaired by host cell enzymes, leading to stably integrated proviral DNA [4]. The IN proteins of different viruses exhibit distinct preferences for integration loci, but DNA sequence *per se*, does not seem to be a major determining factor [5-8]. For HIV-1, and likely other integrases and transposases, interaction with host chromatin-bound proteins plays an important role in such selection [9,10]. Therefore, both the catalytic activities and protein-protein interactions of IN are critical for its function.

The development by Katzman *et al*. [11] of an oligodeoxynucleotide-based assay to study the biochemical properties of IN proteins *in vitro *was an important milestone in the field (Figure 1B). In this assay, a short, radioactively labeled DNA duplex comprising the sequence of either or both viral DNA ends is incubated with the cognate IN protein. The processing and subsequent joining of the labeled strand to self or other targets DNAs, can then be followed by electrophoresis on sequencing gels, allowing all of the substrates and products to be identified [12,13]. Since these original reports, numerous variations on this assay theme have been developed, including the substitution of reporters other than radioactivity, and addition of modifications (e.g., biotin) that facilitate isolation of the products. Such variations have allowed for the development of high throughput screens for inhibitors, and have facilitated the analysis of each step in the reaction. Nevertheless, for many research laboratories, radioactive substrates and gel assays are still employed, despite the fact that such methods are laborious, time-consuming, and not well-suited for kinetic analyses or investigations that require the testing of a large number of proteins or reaction parameters. This problem was alleviated partially through the development of a fluorescence anisotropy assay, to study the DNA binding and processing activities of IN [14].

More recently, we have developed a rapid, sensitive, and simplified fluorescence-based assay to study the joining activity of IN proteins in solution. In this report we describe and validate the assay, and illustrate its utility in a comparison of the joining properties of ASV and HIV-1 integrase, as well as their responses to inhibitory compounds. A preliminary report of this method, together with detailed protocols for fluorescence-based DNA binding and processing assays, has been published [15].

## Methods

### Protein preparation

The ASV and HIV-1 IN proteins used in these studies were purified from the soluble fraction of bacterial lysates after expression of untagged versions of the proteins from plasmid vectors. Similar procedures were employed for both proteins and no detergents were used during the purification, as previous reports have noted that they can affect the multimeric state and the Mg^++^-dependent activities of these enzymes [16].

The wildtype ASV IN protein used in these experiments was expressed and purified as follows: Bacterial cells, BL21 [DE3], containing the plasmid pET29 that expresses wildtype ASV IN (Schmidt-Rupin B strain), were induced to express IN, harvested from 1 liter of Luria broth culture and stored frozen. The frozen cell pellets were thawed and resuspended in lysis buffer (50 mM Tris-Cl pH 7.5, 4 M NaCl, 1% thiodiglycol, 0.1 mM EDTA, 10% glycerol) at 0.1–0.2 g of wet cells/ml. The cells were lysed by two passes through a French Pressure cell at 20,000 psi. The lysate was then subjected to an overnight polyethylene glycol (PEG-8000)-dextran phase separation at 4°C to remove DNA, and the PEG phase was adjusted to 0.2 M salt concentration by conductivity prior to batch purification on phospho-cellulose (Whatman P11). After washing, IN was eluted with phospho-cellulose elution buffer (50 mM Tris-Cl pH 7.5, 1.2 M NaCl, 1% TDG, 0.1 mM EDTA, 10% glycerol). The fractions containing IN were identified by SDS-polyacrylamide gel electrophoresis (PAGE) and pooled. Aliquots were diluted five-fold to reduce the final salt concentration to 0.2 M, and immediately applied to a 5 ml HiTrap heparin column equilibrated with heparin binding buffer (50 mM Tris-l pH 7.5, 0.2 M NaCl, 10% glycerol). Following a wash step, the bound protein was eluted with a gradient from 0.2 to 1.2 M NaCl in the same buffer. The fractions containing IN were again identified by SDS-PAGE, pooled, concentrated, and dialyzed against three changes of 1 liter 50 mM Hepes pH 8.1, 0.5 M NaCl, 1% thiodiglycol, 0.1 mM EDTA, 1 mM dithiothreitol (DTT), 40% glycerol. Following dialysis, aliquots were flash frozen in liquid nitrogen at ~1–2 mg IN/ml. We note that wildtype ASV IN can also be purified using the method described below for HIV-1 IN, with no significant difference in yield or specific activity.

The HIV-1 IN protein was expressed and purified as follows: Bacterial cells, BL21 [DE3], containing the plasmid pET29 that expresses wildtype HIV-1 IN (NY5 strain), were induced to express IN, harvested from 1 liter of Luria broth culture and stored frozen. The frozen cell pellets were thawed and resuspended in lysis buffer (25 mM BisTris-HCl pH 6.1, 1 M NaCl, 1 M urea, 0.1 M imidazole, 5% glycerol with protease inhibitors (aprotinin, leupeptin, phenylmethyl sulfonyl fluoride, and pepstatin) at 0.13 g of cells/ml. The cells were lysed by passage through a French Pressure cell as above, and the lysate was then sonicated for 30 s. The preparation was subjected twice to centrifugation for 30 min at 12,000 × *g*. Solid NaCl was added to the supernatant fraction to bring it to 4 M concentration, and it was then applied to a 22 ml methyl hydrophobic interaction chromatography column (Biorad) equilibrated with HIC Buffer A (25 mM BisTris-HCl pH 6.1, 1 M urea, 4 M NaCl, 0.1 M imidazole, 5% glycerol, and 6 mM 2-mercaptoethanol). Following a brief wash, the bound protein was eluted with a linear gradient to HIC Buffer B (contents identical to HIC Buffer A with the exception of 0.2 M NaCl). The fractions containing IN were identified by SDS-PAGE. Protease inhibitors were again added to these fractions and they were then pooled in preparation for the second column step. Aliquots of this pool were diluted to reduce the final salt concentration to 0.2 M, using a buffer containing 50 mM BisTris-HCl pH 6.5, 1 M urea, 0.1 M imidazole, 5% glycerol with 6 mM 2-mercaptoethanol. This solution was immediately applied to a 5 ml HiTrap heparin column equilibrated with Heparin Buffer A (25 mM BisTris-HCl pH 6.1, 1 M urea, 0.2 M NaCl, 0.1 M imidazole, 5% glycerol and 6 mM 2-mercaptoethanol). Following a wash step, the bound protein was eluted with an exponential gradient of 0.2 to 1.2 M NaCl in the same buffer. The fractions containing IN were identified by SDS-PAGE, pooled, concentrated, and dialyzed against three changes of 1 liter 25 mM BisTris-HCl pH 6.1, 1 M NaCl, 1% thiodiglycol, 1 mM dithiothreitol (DTT), 40% glycerol. Following dialysis, aliquots were flash frozen in liquid nitrogen at ~1–2 mg IN/ml.

### DNA substrates

Viral DNA (donor) oligodeoxynucleotides with a covalently attached 6-carboxyfluorescein (6-FAM) were purchased from Integrated DNA Technologies (Coralville, IA), and purified by Tris-borate urea denaturing polyacrylamide gel electrophoresis. The efficiency of labeling was quantified by comparison of the absorbance at 260 nm with the peak absorbance of the fluorophore (495 nm for 6-FAM). The labeled oligodeoxynucleotides were annealed with unlabeled complementary oligodeoxynucleotides to obtain viral donor oligodeoxynucleotide duplexes. Complementary strands of the target oligodeoxynucleotide containing biotin at their 3'-ends were synthesized and purified in the Fox Chase DNA Synthesis Facility. These were then annealed to obtain a 27 bp duplex with single nucleotide overhang on each 3'-end to which biotin was attached.

### Fluorescence assays for enzymatic activities

**Figure 2:**
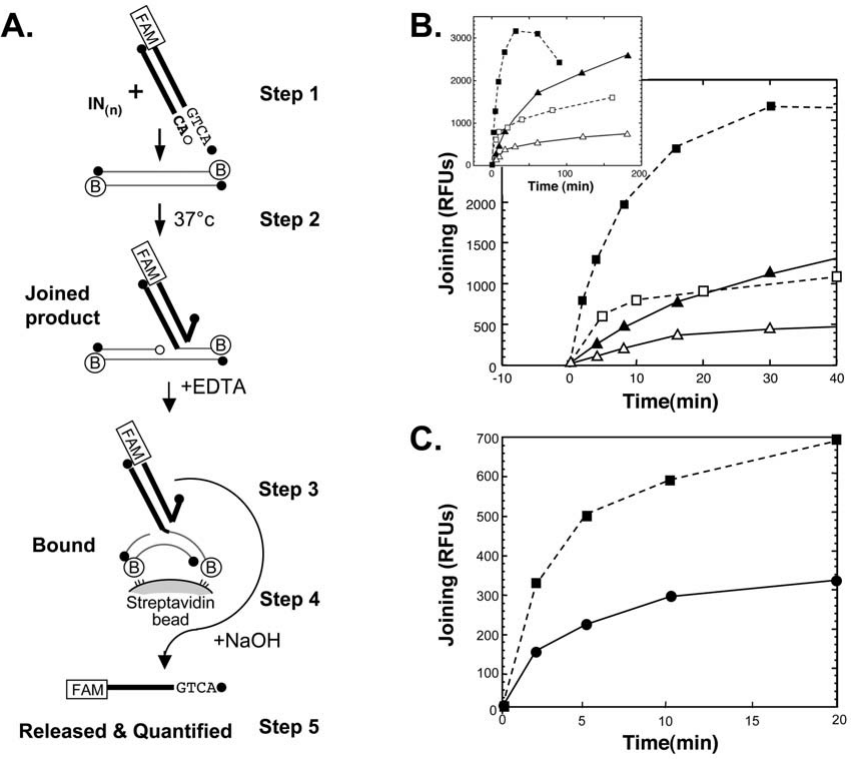
**Moderate-throughput solution assay for integrase joining activity**. Panel A. Principles of a solution assay to measure integrase joining activity by fluorescence. Labeling and symbols are as in Figure 1. FAM stands for carboxyfluorescein labeled DNA, a circle with B denotes a biotin modified 3' end in the target oligodeoxynucleotide. Panel B. Comparison of HIV-1 and ASV IN joining activities in Mg^++ ^and Mn^++^. The dashed lines with squares show the activity of ASV IN and the solid lines with triangles show the activity of HIV-1 IN expressed as RFUs versus time. Filled and open symbols represent activity in Mn^++ ^and Mg^++^, respectively. The inset shows results from the same experiment, after 40 min. and up to 180 min. incubation. Panel C. Comparison of the joining activity of ASV IN with the recessed versus the blunt-ended donor oligodeoxynucleotides in the presence of Mg^++ ^(recessed donor oligodeoxynucleotide, dashed line with filled squares; blunt-ended donor oligodeoxynucleotide, solid line with filled circles).

Processing activity was measured using fluorescence-anisotropy [14,15]. The fluorescence intensity assay for joining (Figure 2A) was performed as follows:

### Steps 1–2. Preincubation and reaction conditions

**Figure 3:**
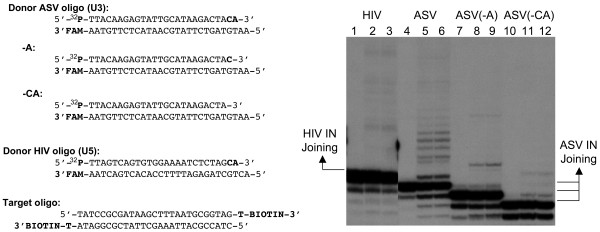
**Joining activity confirmed with gel electrophoresis**. Left, sequences of the donor oligodeoxynucleotides used in the joining assay. The location of carboxyfluorescein (FAM), 5' radioactive ^32^P, and 3' biotin are shown. The -A substrate removes only the A of the conserved CA dinucleotide while the -CA substrate removes both residues. Right, lanes 1 through 3 show HIV-1 IN joining activity on its substrate after 0, 60, 120 min of incubation, respectively. Lanes 4 through 6, 7 through 9, and 10 through 12, show ASV IN joining activity after 0, 15, 30 min of incubation.

The double stranded, 6-FAM-labeled viral oligodeoxynucleotide (donor substrate) was mixed with IN and the metal cofactor, and the mixture was left on ice for 15 min. The biotin-conjugated, double stranded target oligodeoxynucleotide was then added, and the mixture left on ice for an additional 15 min, after which it was transferred to a waterbath at 37°C and incubated for the desired period. The total reaction volume was 20 μl. We determined the optimal ratio of IN:viral oligodeoxynucleotide:target oligodeoxynucleotide, to be 4:1:6, and this ratio was used to test the potency of the inhibitors. These reactions contained 1 μM IN, 0.25 μM 6-FAM-labeled viral oligodeoxynucleotide (26nt/28nt recessed duplex), 1.5 μM biotin-conjugated target oligodeoxynucleotide duplex, 5 mM DTT or 2 mM mercaptoethanol, 10% DMSO, 25 mM Hepes, pH 7.5 (at 37°C) with 10 mM MnCl_2 _(Fisher, Certified ACS) or 10 mM MgCl_2_, (Fisher, Certified ACS) and ionic strength ≤ 100 mM NaCl equivalents. The reactions were stopped by the addition of 10 μl of 30 mM EDTA. For the comparisons described in Figures 2 and 3, we used a slightly sub-optimal ratio of 2:1:6 that allowed for the detection of both increases and decreases in joining activity. These reactions contained 1 μM IN, 0.50 μM 6-FAM-labeled viral oligodeoxynucleotide, and 3.0 μM biotin-conjugated target oligodeoxynucleotide.

### Step 3. Product capture

A 96 well filter plate (Pall Life Sciences; AcroPrep 96 filter plate, 0.45 μm GHP membrane, 350 μl/well, PN 5030) was prepared for use by adding 50 μl of a 1:1 slurry of streptavidin agarose beads to each well (Invitrogen; streptavidin agarose, sedimented bead suspension, PN S951). The assay reactions were transferred to the wells, and incubated at room temperature for 30 min (with gentle shaking at 5 min intervals) to allow the biotin-conjugated target and joined products to bind to the beads. The wells were then washed 10 times with 200 μl Wash Buffer (1× PBS, 0.05% SDS, 1 mM EDTA) using a vacuum manifold (Pall Life Sciences; Multi-well Plate Vacuum manifold, PN5017). In some cases, the last wash was also collected by centrifugation into a reader plate and analyzed to confirm that all of the unbound, unjoined FAM-labeled viral oligodeoxynucleotide had been removed.

### Step 4. Probe release

The viral oligodeoxynucleotide strand that included the 6-FAM probe was dissociated from the bound product by denaturation via addition of 150 μl of freshly prepared 50 mM NaOH to each well. The plate was then left at room temperature for 5 min. The soluble fractions were collected by centrifugation (2,000 × g/10 min) into a black, round bottom 96 well plate (Costar, storage plate, PN 3356).

### Step 5. Detection and analysis of the released product data

**Figure 4:**
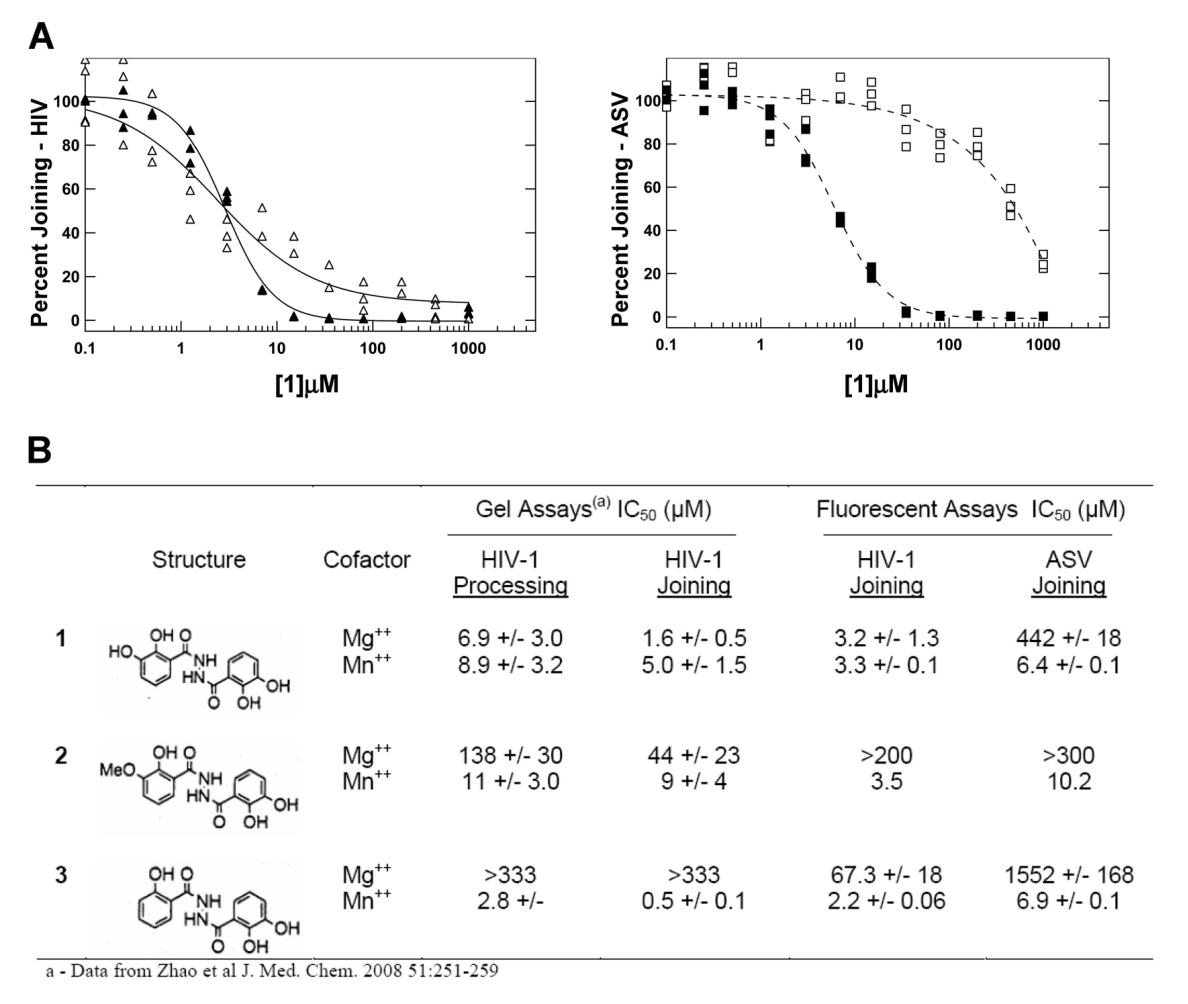
**Tests of the metal cofactor effects of HIV-1 IN inhibitors on HIV-1 and ASV IN joining activities**. A. Dose response curves showing the joining activities of HIV-1 and ASV IN (at 1 μM concentration) as a function of increasing concentration of compound **1**. Triplicate data are plotted for each inhibitor concentration and the curves show non-linear regression fitting of the data using Visual Enzymics software. The solid and open triangles represent HIV-1 IN activity in the presence of Mn^++ ^or Mg^++ ^cofactors, respectively. The solid and open squares represent ASV IN activity in the presence of Mn^++ ^or Mg^++ ^cofactors, respectively. B. Comparison of the IC_50 _values obtained by gel and solution based methods. The structure of the inhibitors is shown to the left of the table. Previously published values for IC_50_s with HIV-1 IN are shown on the left, while values on the right for both HIV-1 and ASV IN were obtained with the solution assay described here. The latter values were determined from non-linear fitting of the triplicate data to a four parameter sigmoidal dose response equation, with the standard error of the fit shown for compounds **1 **and **3**. Data for compound **2 **are from a single experiment.



The wells were read using a Tecan GENois Pro fluorescent microplate reader equipped with Magellan Standard V5.03 software (Tecan Austria GmbH, Salzburg, Austria) set to the fluorescence intensity mode. In this instrument the excitation of 6-FAM is at 485 nm and the emission is measured at 535 nm. The data from the plate scanner are expressed as relative fluorescence units (RFUs). The experimental RFU readings, including the data from the background wells and the controls were transferred to the Visual Enzymics (Softzymics, Princeton, NJ) module running with Igor Pro (Wavemetrics, Inc.) graphing software. In the experiments described in Figure 4, the IC_50 _values were determined from non-linear fitting of the triplicate data to a four parameter sigmoidal dose response equation:

where A is the activity at maximal inhibition, B is the activity in the absence of inhibitor, X is the inhibitor concentration, C is the IC_50 _value, and D is the Hill coefficient. The Hill coefficient, which is proportional to the slope of the sigmoidal curve, reflects the cooperativity and the tightness of binding of the inhibitor to the enzyme. All four parameters are fitted, and the standard error and Chi squared goodness of fit statistics confirm adequate data quality. The data are then plotted as percent joining activity to compare the various enzymes, metals, and inhibitors used.

### Standard radioactive gel assays

The same viral donor DNAs were assembled after the strand to be processed was ^32^P-labeled at its 5' end. These strands were then annealed with complementary oligodeoxynucleotides that were labeled with 6-FAM, as described for the fluorescent assay above. The target DNA and reaction conditions followed those described for the fluorescent assay. The products were separated by electrophoresis in a Tris-borate-urea 20% polyacrylamide gel and quantified using a Fuji phosphorimager. The processed products migrated below the substrate bands, and the joined products migrated in a series of bands above the substrates.

## Results

### Principles of the fluorescence-based joining assay

This assay employs a short DNA duplex (e.g., 18–28 base pairs) comprising the sequence at the end of one or the other viral LTR, hereafter called the donor oligodeoxynucleotide. As illustrated in Figure 2A, the 3' end of the strand complementary to that which is cleaved by IN is labeled with carboxyfluorescein (6-FAM). To study only the joining reaction, the donor oligodeoxynucleotide has a recessed CA end, as would normally be produced in the processing reaction. The details of the assay, provided in Methods, are outlined briefly in Figure 2A. In step 1, the donor oligodeoxynucleotide is mixed with IN and the required divalent metal cofactor (Mn^++ ^or Mg^++^) in a suitable buffer on ice. The target oligodeoxynucleotide, which contains biotin at both 3' ends, is then added in molar excess over the donor. In step 2, the mixture is incubated at 37°C for the desired period, after which catalysis is stopped by the addition of an excess of EDTA. In step 3, the reaction is transferred to a well in a 96 well filter plate that contains a slurry of streptavidin agarose beads. This mixture is left at room temperature for 30 min and shaken gently at 5 min intervals. In step 4, the beads are washed thoroughly with suction applied in a multi-well plate vacuum manifold. A solution of 50 mM NaOH is then added to each well to denature the DNA and the mixture left for 5 min at room temperature. In step 5, the solution containing the released FAM-labeled donor single strands is collected by centrifugation into a 96 well plate. The fluorescence of the FAM-labeled donor in each well is recorded in a plate reader.

During optimization studies, we measured the relative activities of ASV and HIV-1 IN in the presence of both cofactors, and observed an increase with increasing divalent metal concentration to a maximum at approximately 15 mM for both proteins. We note that higher metal concentrations promote the non-specific endonuclease of IN proteins and can raise the ionic strength to inhibitory levels. To avoid these problems and to establish uniform conditions for our comparisons we chose the close to optimum concentration of 10 mM to measure the joining activities of these two proteins.

The pH-dependence of both the processing and the joining reactions with ASV and HIV-1 IN proteins in the presence of either Mn^++ ^or Mg^++ ^was also determined. The results from our joining assays indicated that with Mn^++ ^as cofactor, both enzymes exhibit activity maxima in the range of pH 7–7.5; maxima for processing with Mn^++ ^are higher, at pH 8.1 for both enzymes. Rather different results were obtained with Mg^++ ^as cofactor. In this case, optima for ASV IN were in the range of pH 8–8.5 for both processing and joining, whereas the optima for HIV-1 IN were substantially lower, pH 7 for processing and pH 6.5 for joining, although the ranges were fairly broad.

### Side-by-side comparison of ASV and HIV-1 IN joining activities with Mn^++ ^or Mg^++ ^as the metal cofactor

Although the physiologically relevant cofactor for retroviral IN activity *in vivo *is believed to be Mg^++ ^[17], both ASV and HIV-1 IN proteins are reported to be more active with Mn^++ ^as the cofactor. For comparison of the activities of these proteins, as donor oligodeoxynucleotides we used sequences from the U3 (ASV IN) and U5 (HIV-1 IN) LTRs, because previous studies have shown that the enzymes are most active with these DNA ends [18,19]. The ratio of IN to donor DNA was 2:1 as preliminary experiments indicated that this was close to the optimum for both enzymes. To accommodate the differences noted above, the ASV IN reactions were run at pH 8.0 and the HIV-1 IN reactions at pH 7.3 in which joining was expected to be close to optimal with both metals. When analyzed under these conditions, the initial rate for joining by ASV IN with Mn^++ ^as the cofactor was 6.7 times faster than HIV-1 IN, and with Mg^++ ^it was 5.3 times faster (Figure 2B). With both enzymes, the initial rates in the presence of Mg^++ ^were 30 to 40 percent of that with Mn^++^. In both cases, the initial "burst" of product in the presence of Mg^++ ^leveled off rather quickly (within 5 to 15 min), and then product continued to increase at a much reduced rate. A similar response was observed in the presence of Mn^++^, but the rate of the second phase was higher. In both cases, the initial bursts are likely to represent product from donor-enzyme complexes formed during the preincubation step (Figure 2A, Step 1). The subsequent, reduced rates reflect the slow turnover characteristic of these enzymes, and competition between donor and target oligodeoxynucleotides for enzyme binding in subsequent rounds of catalysis. Similar effects have been noted in studies of joining by ASV IN [20]. In the case of ASV, we observed an apparent decrease in the amount of product after 50 min (Figure 2B, inset), which may be explained by the increased non-specific nuclease activity of this enzyme in the presence of Mn^++ ^[21].

The joining assay can also be used with non-recessed, blunt-ended donor oligodeoxynucleotides. However, such a donor end must first be processed by IN before it can be joined to the target oligodeoxynucleotide. Figure 2C shows a comparison of the joining activities of ASV IN with recessed and blunt-ended donor DNAs, in the presence of Mg^++^. The initial rate with the blunt ended donor is less than half that observed with the recessed end donor, indicating that the overall reaction rate is limited substantially by processing. Guiot *et al*. [14] have shown that the rate of processing by HIV-1 IN is also relatively slow.

### Joining activity is confirmed by polyacrylamide gel electrophoresis

To verify that joining has indeed taken place in the context of this assay, we added a radioactive (^32^P) label to the 5' end of the donor strand to be joined, and then analyzed the products using gel electrophoresis. The donor and target oligodeoxynucleotides in these reactions were otherwise identical to those used in our standard fluorescence assay (Figure 2B), and the sequences are shown in Figure 3. As controls, we also prepared and tested radioactively labeled ASV donor oligodeoxynucleotides that lacked either the A of the conserved CA, or both nucleotides. Results from two time points were analyzed in each case. As illustrated in the gel data (Figure 3 right), joined products were detected in both the HIV-1 and ASV IN reactions with the respective donor oligodeoxynucleotides, in the same relative proportions as determined in the fluorescence assay. As expected from numerous previous studies, severely reduced joining was observed with the donors that lacked one or both of conserved, terminal CA dinucleotides.

Table 1 shows a comparison of signal-to-background ratios calculated for the same time points in the experiments of Figure 2B and Figure 3, as well as from previous gel analyses (not shown). These data indicate that the fluorescence-based joining assay is approximately 20–30 times more sensitive than the gel assay in reactions catalyzed by either enzyme in the presence of Mn^++^. With Mg^++ ^as cofactor, the increase in sensitivity is at least 10-fold for HIV-1 IN, and approximately 20-fold for ASV IN. Data from the fluorescence joining analyses in Figure 2B were also used to calculate the signal to noise ratio, which is a more statistically significant measure of the quality of an assay, as it includes standard deviation of the background as a parameter [22]. Values obtained for ASV IN were 169 with Mn^++ ^(15 min) and 205 with Mg^++ ^(30 min).

**Table 1:** Comparison of signal to background ratios for fluorescence-based and gel joining assays

		**HIV-1 IN**		**ASV IN**	
Cofactor	Assay	60'	120'	15'	30'
					
Mn^++^	a. Fluorescence	61	78	126	155
	b. Gel	2.5	3.4	4.2	4.5
	Fold Difference (a/b)	24.4	23	30	34

Mg^++^	c. Fluorescence	21.4	26.5	26.3	31.3
	d. Gel*	--	2.6*	--	1.3*
	Fold Difference (c/d)		10		24

Signal-to-background ratios were calculated by dividing the values obtained in the presence of IN by those obtained in the absence of IN in the released fluorescent product (Figure 2B) or the relevant region of the gel (Figure 3). Ratios marked with an asterisk are from previous gel assays (not included), with ASV IN at the indicated time and HIV-1 IN at 180'.

### Use of the fluorescence-based joining assay for identification of HIV-1 IN inhibitors that are effective against ASV IN

We were also interested in evaluating the utility of the fluorescent assay for determining IC_50 _values for integrase inhibitors. In this context, Zhao *et al *[23] recently reported the development of a number of novel metal chelating inhibitors of HIV-1 IN, several of which were found to be effective in blocking both processing and joining in the presence of either Mn^++ ^or Mg^++^. Of special interest for our analyses, was a related series of 2,3-dihydroxybenzoic acid hydrazides (Figure 4B) [23-25]. Compound **1**, is a symmetrical molecule reported to block both the processing and joining activities of HIV-1 IN, with either metal cofactor. In compound **2**, one hydroxyl on the left benzoyl ring is substituted with a methoxyl group, a change that was reported to have little effect on the inhibitory potency for HIV-1 IN with Mn^++^, but resulted in reduced potency with Mg^++^. Removal of the same hydroxyl to produce compound **3 **also had little effect in Mn^++^, but the potency in Mg^++ ^was reduced even further. We tested these compounds for cofactor-dependent activity against both HIV-1 and ASV IN proteins at 1 μM concentration, using our fluorescence joining assay (Figure 4).

The concentration dependence for compound **1 **inhibition of joining by HIV-1 and ASV IN proteins is shown in Figure 4A. As reported previously [23], HIV-1 IN is almost equally sensitive to this compound in the presence of either metal cofactors. Similar inhibition is seen for ASV IN with this inhibitor in the presence of Mn^++^, but ASV IN is much more resistant to this compound in the presence of Mg^++^. It is noteworthy that with both enzymes the slopes of the dose response curves is steeper in the presence of Mn^++ ^(Hill coefficient of 2–2.5) than Mg^++ ^(0.6–1.3). This is indicative of a greater cooperativity of inhibitor binding with the Mn^++ ^cofactor, and is consistent with results from previous studies of this class of inhibitors [17]. The Z' factor [22] calculated from the assays performed in these experiments was 0.7, which represents a "good" value for screening fitness.

A summary of the IC_50 _values calculated for all three inhibitors is shown in Figure 4B. The results from the fluorescence joining assays with HIV-1 IN generally correspond to those reported for the gel assays, thus validating its utility for such studies. These analyses show that ASV IN is slightly (~2–5-fold) less sensitive than the HIV-1 enzyme to inhibition by these compounds in the presence of Mn^++^. From these results, it appears that in the presence of this metal cofactor, all three compounds interact with structural elements that are conserved in these two IN proteins, and this interaction inhibits the joining reaction. Results with compound **1 **indicate that this inhibitor is able to discriminate between the two proteins in the presence of Mg^++^.

## Discussion

### The joining assay

In this report we describe a simplified assay measuring the joining activity for retroviral integrases in solution. The assay offers several advantages over the gel analyses used in many laboratories. Limitations of the gel assays include the length of time needed to separate and quantify the products and relatively low sensitivity. The latter problem derives from the fact that the ligated products detected in this assay are of different sizes and therefore spread through a large portion of the gel (see Figure 3), such that backgrounds can be a problem. In the solution assay we have developed, the uniformly sized, non-ligated viral donor strand is scored in each reaction. Our signal to background calculations (Table 1) indicate that the fluorescence assay is approximately 10–30 times more sensitive than the standard gel assay for measuring this activity. In addition, the assay is much faster than gel analysis and numerous samples can be handled with relative ease.

The assay described here builds upon features introduced by several investigators in earlier efforts to facilitate analysis of the joining reaction both for biochemical studies and identification of inhibitors. The use of biotin in combination with streptavidin-coated plates or beads, as well as magnetic beads, to select joined products has been described previously in our lab and others [20,26-29]. Reporters for the recombination products have included radioactivity [20,26] and digoxygenin plus a conjugated antibody that allows amplification of the signal [27-29]. However, most of these previously described methods require more steps than our assay and, in some cases, the reactions are designed to take place on a solid surface [29-31], which is well-suited for high throughput screening of inhibitors but not for biochemical analyses. Furthermore, the shelf life of the fluorescent substrates is not limited by radioactive decay.

For our standard assay, we chose carboxyfluorescein as a reporter because the signal can be detected easily and directly in a plate reader. This reporter was used extensively by Deprez and coworkers in the development of fluorescence-based assays for DNA binding and processing by IN [14,32], which we have found to be extremely useful. Together with our joining assay, they provide a convenient fluorescence-based suite of methods with which to analyze the properties of IN proteins using the same detection system [15]. However, if necessary, the sensitivity of the assay could be increased further by use of other reporters such as radioactivity or digoxygenin plus antibody for amplification. Finally, the assay can be adapted for measuring disintegration, i.e. reversal of the joining reaction (Figure 1B) [33,34].

The novel elements of our joining assay are 1) the placement of the reporter on the donor strand complementary to that which is actually joined, and its dissociation from the bound product and 2) the attachment of biotin to the 3' ends of both strands of the target DNA. The first feature allows for better detection of the reporter, as its signal is obscured when retained on agarose beads. After developing this protocol we discovered that a similar strategy was employed by Landgraf *et al*. [35] in development of a quantitative assay for PCR products. The advantage of having biotin on both strands of the target DNA is that products of joining to either target DNA strand will be captured, thereby improving sensitivity. At present this assay is suitable for moderate throughput applications, as reactions are run in separate tubes. This is adequate for routine laboratory research, but the method could be modified for higher throughput and inhibitor screening, if desired. In the latter case, a reporter other than carboxyfluorescein might be more useful, as candidate inhibitors that exhibit intrinsic fluorescence could increase the background.

### The similarities and differences in the cofactor responses with HIV-1 and ASV IN

A side-by-side detailed comparison of the cofactor-dependent joining activities of purified HIV-1 and ASV IN proteins used to illustrate the utility of this new assay revealed a number of similarities, as well as some notable differences. Although Mg^++ ^is likely to be the biologically-relevant cofactor, the initial rates of joining by both isolated enzymes with Mg^++ ^are less than half the rate, with Mn^++^. Both enzymes also exhibit a similar pH optimum (7–7.5) in the presence of Mn^++^. However, with ASV IN, the optimum for joining in Mg^++ ^is somewhat higher (pH 8–8.5), and with HIV-1 IN lower (pH 7–6.5) than with Mn^++^. The reason for these differences is unknown, but these data suggest that the two metals are bound differently by these enzymes, and/or that the microenvironment for binding Mg^++ ^is not the same in the two proteins. Finally, the rate of joining by ASV IN is 6–7 fold faster than HIV-1 IN in the presence of either metal cofactor.

We also demonstrated the utility of the joining assay for screening inhibitors, by testing the potency of a related series of compounds known to block HIV-1 IN, on the activities of both IN proteins. The IC_50 _values obtained with HIV-1 IN were similar to those previously reported with a gel assay, despite the fact that our assay conditions are quite different [36]. We also observed that, like HIV-1, ASV IN was sensitive to inhibition by all three compounds in the presence of Mn^++^, although the IC_50 _values were approximately 2 to 5 times higher with this enzyme. This finding is consistent with the notion that Mn^++ ^is bound in similar ways by these two proteins. Inhibitor **1 **was of special interest as it was reported to be equally effective with HIV-1 IN in the presence of either metal cofactor, and those results were also confirmed by our assay. Our finding that this compound was ineffective against ASV IN in the presence of Mg^++^, further supports the notion that the determinants for binding of Mg^++^, or a Mg^++^-inhibitor complex, are different in the two enzymes.

## Abbreviations

The abbreviations used are: IN: retroviral integrase; HIV-1: human immunodeficiency virus; ASV: avian sarcoma virus; 6-FAM: 6-carboxyfluorescein.

## Competing interests

The authors declare that they have no competing interests.

## Authors' contributions

MDA supervised the work and data analysis, and contributed to writing and editing the manuscript. JC designed the assay and performed some of the preliminary experiments. GM conducted all of the optimization studies and performed all of the assays and some of the calculations included in the manuscript. XZZ synthesized and tested the HIV-1 inhibitors under the supervision of TRB, Jr. AMS provided overall direction and had primary responsibility for writing and finalizing the manuscript, which all authors have read and approved.
